# Trade, protectionism, and climate: Assessing analytical capacity in a changing policy landscape

**DOI:** 10.1016/j.isci.2026.117132

**Published:** 2026-08-13

**Authors:** Paola Rocchi, Valentina Bosetti, Baptiste Boitier, Lorenza Campagnolo, Alessia Campolmi, Maksym Chepeliev, Chiara Forlati, Panagiotis Fragkos, Lukas Hermwille, Alexandros Nikas

**Affiliations:** 1 CMCC Foundation - Euro-Mediterranean Center on Climate Change, Milan, Italy; 2 RFF-CMCC European Institute on Economics and the Environment, Milan, Italy; 3 Department of Economics, Bocconi University, Milan, Italy; 4 SEURECO - Société Européenne d’Économie SARL, Paris, France; 5 Department of Economics, University of Verona, Verona, Italy; 6 Center for Global Trade Analysis, Department of Agricultural Economics, Purdue University, West Lafayette, IN, USA; 7 School of Economic, Social and Political Sciences, University of Southampton, Southampton, UK; 8 E3-Modelling S.A., Athens, Greece; 9 Wuppertal Institute for Climate, Environment and Energy, Wuppertal, Germany; 10 School of Electrical and Computer Engineering, National Technical University of Athens, Athens, Greece

**Keywords:** Trade-climate nexus, Trade policy dynamics, Environmental outcomes of trade, Modeling frameworks and enhancements, Data and scenario design priorities

## Abstract

Strategic, competitiveness-driven, and protectionist trade measures are reshaping global trade, while climate commitments face mounting urgency. This evolving policy landscape requires renewed scrutiny of the trade-climate nexus, as governments depart from liberalization and adopt interventions that affect trade and production patterns. This perspective offers a framework that integrates fragmented insights on trade-climate interactions and applies them to current policy developments. By mapping how trade influences emissions and identifying the conditions shaping environmental outcomes, the paper clarifies when and how different trade regimes may hinder or support climate objectives. Building on this foundation, the paper assesses whether existing data, modeling frameworks, and analytical tools are equipped to capture these dynamics and identifies critical gaps that limit current assessments. Finally, the paper outlines priorities for improving data integration, modeling approaches, and scenario design, proposing a forward-looking research agenda to strengthen the analytical basis for evaluating the climate implications of evolving trade regimes.

## Graphical abstract

**Figure:**
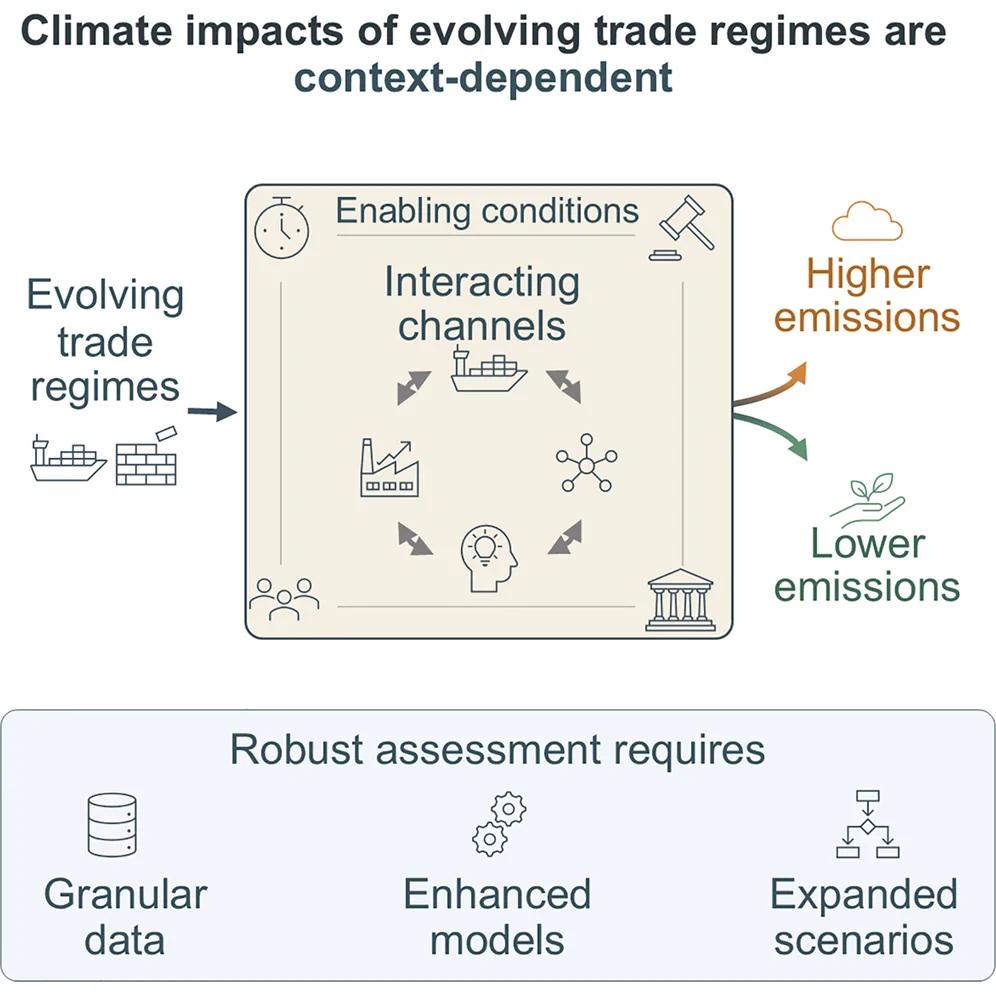

## Teaser

Environmental science; Environmental policy; Social sciences

## Introduction

As strategic trade interventions gain momentum^1^^,^^2^ and climate commitments face mounting urgency, the intersection of trade and climate policy demands renewed scrutiny.^3^ Recent shifts in economic governance suggest a departure from decades of liberalization, with governments increasingly adopting measures to boost domestic competitiveness^4^ or to restrict imports.^5^ While trade-restricting measures (tariff and non-tariff barriers, import and export bans, etc.) directly limit market access, industrial policies aiming to strengthen domestic competitiveness and strategic autonomy (subsidies, local content requirements, etc.) also indirectly affect trade (see Box 1 for illustrative examples). These measures, by reprioritizing domestic competitiveness over global cooperation, risk weakening collective action needed to achieve common climate objectives.^6^ This may, in turn, increase uncertainty around emissions pathways and reshape patterns of cooperation, regionalization, and technological competition.

Box 1 **Illustrative examples of recent shifts in trade governance**

• **United States**: Tariffs, trade agreement renegotiations, and the Inflation Reduction Act support domestic clean-technology industries and strategic autonomy, with implications for trade flows and technology deployment.^4^^,^^5^
• **European Union**: The Carbon Border Adjustment Mechanism (CBAM), industrial subsidies, and trade measures targeting clean technologies, including “union origin” content requirements increasingly link climate policy with industrial competitiveness and trade strategy.^7^^,^^8^
• **China and India**: Domestic production mandates and support for key green sectors aim to strengthen industrial capacity and shape global supply chains.^9^

In this perspective, we argue that this evolving policy context calls for a scrutiny of how trade-climate interactions are analyzed. More specifically, we argue that trade-climate interactions should be assessed in the context of evolving trade regimes rather than under the implicit assumption of stable, broadly liberalized trade systems. Existing approaches largely rely on such assumptions, which may no longer hold. Hence, the goal of this paper is to explore whether existing analytical tools are well-equipped to capture the mechanisms through which evolving trade regimes shape climate outcomes and to identify key gaps and priorities for improvement.

The paper is structured around three building blocks. First, it develops an integrated conceptual framework of the main channels linking trade and emissions, with the aim of clarifying the mechanisms through which evolving trade regimes may influence emissions and climate mitigation outcomes. While previous papers have reviewed the main channels through which trade affects environmental outcomes,^10^^,^^11^^,^^12^ our contribution lies in synthesizing key insights into a unified framework that captures the complexity of the trade-climate nexus and provides a benchmark for systematic assessment. Second, the paper analyses the extent to which existing analytical tools—data, modeling frameworks, and empirical approaches—are equipped to capture these mechanisms. The purpose is to evaluate the adequacy of current analytical capacity in light of emerging trade and policy dynamics, identifying mismatches between the complexity of the mechanisms at play and the ability of existing tools to represent them, and highlighting critical data, modeling, and methodological gaps. Third, the paper provides recommendations to address these gaps by defining methodological priorities and outlining a forward-looking research agenda in a rapidly changing policy landscape.

## Trade-climate linkages: Channels, dynamics, and enabling conditions

**Figure 1:**
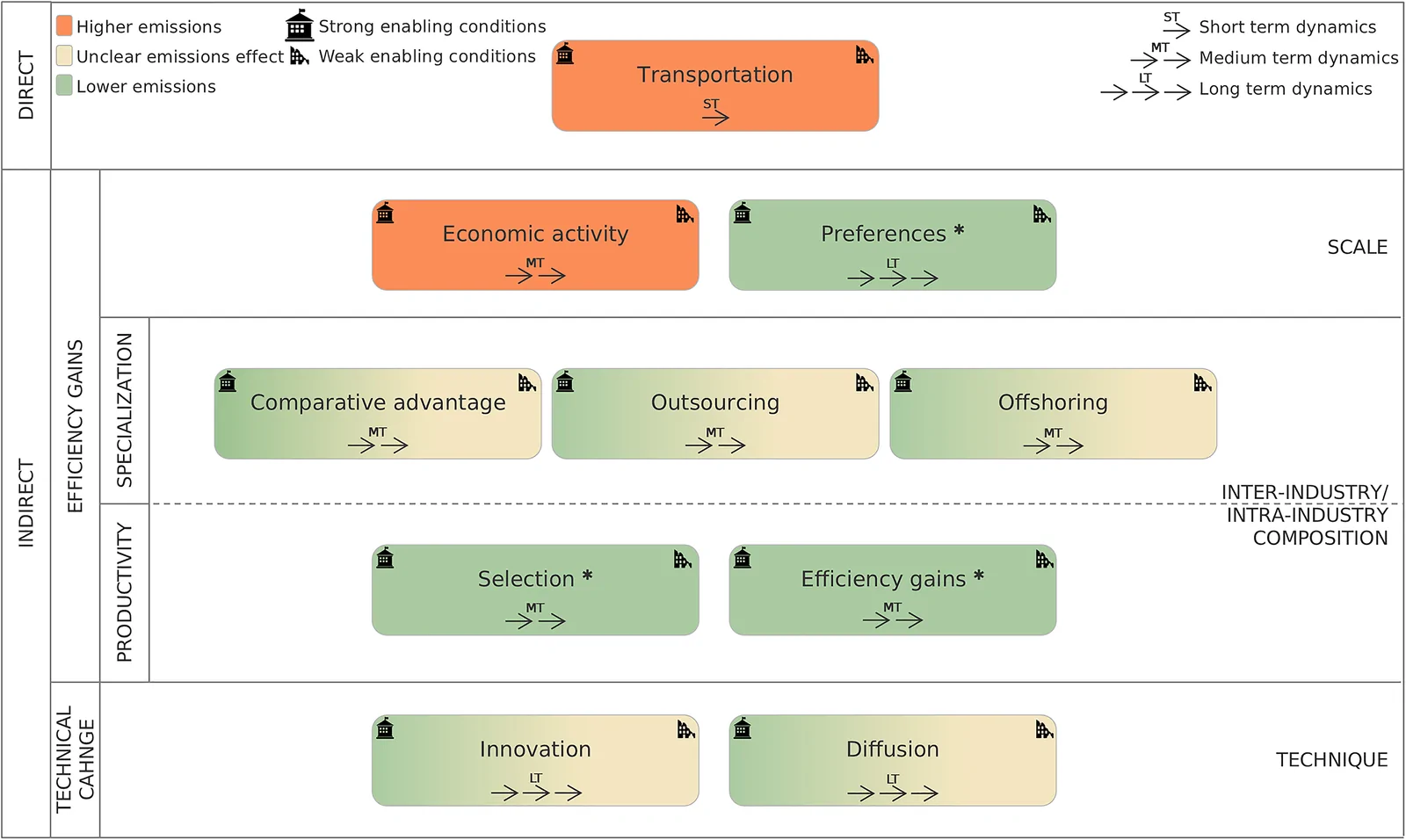
Key channels linking trade to emissions, their temporal dynamics, and the role of enabling conditions for trade’s impact on GHG emissions The direction of impacts refers to the expected emissions effect of each individual mechanism considered in isolation. ^✱^Climate benefits are theoretically expected, but empirical evidence supporting this channel is mixed or context-dependent. Source: own elaboration.

To understand how trade and climate interact, we first identify the main channels through which this relationship unfolds and the factors that shape it. Trade affects the environment through multiple interconnected mechanisms, some direct (e.g., transport emissions), others indirect (changes in production structure and scale, consumption, firm behavior, or innovation). These effects interact, amplifying or offsetting each other in context-dependent ways. This section provides a conceptual overview of the channels through which trade influences climate outcomes and their complex interactions across time. It also identifies critical dimensions—regulatory, institutional, and political—that shape the magnitude and direction of their effects. Figure 1 provides a structured overview of the main channels linking trade and emissions, their temporal dynamics, and the enabling conditions that shape their effects. The discussion below follows this structure.

### Key channels linking trade and emissions

As shown in Figure 1, the direct environmental effect of trade is the rise in transport-related emissions. Cross-border movement of goods relies on maritime shipping, aviation, and land transport, all major sources of greenhouse gas (GHG) emissions. Previous research has quantified emissions,^13^^,^^14^^,^^15^^,^^16^^,^^17^ assessed decarbonization pathways across transport modes,^18^^,^^19^^,^^20^ and analyzed systemic feedbacks using integrated assessment models (IAMs) and computable general equilibrium (CGE) models^21^^,^^22^ (Notes S1 and S2, and Tables S1 and S2). As trade openness increases, so do travel distances and freight activity. Protectionism may reduce emissions by lowering trade volumes and shortening traveled distances, though it could also induce inefficiencies or longer routes via trade diversion.^23^

Beyond transport, trade influences emissions indirectly through structural changes in global production. It reallocates economic activity across countries according to comparative advantages and patterns of specialization, with implications for consumption, production, and supply chains. As illustrated in Figure 1, these effects are commonly captured through three broad mechanisms: the scale, the composition (both inter- and intra-industry), and technique effects.^24^^,^^25^

The scale effect arises when trade liberalization expands market size,^26^ increasing energy and resource demand and absent strong environmental regulation, raising emissions.^27^ Although income growth may eventually shift preferences and policies toward cleaner outcomes—as suggested by the Environmental Kuznets Curve (EKC)^28^—empirical support remains mixed.^29^ Protectionism may dampen these scale effects by slowing growth, but its implications for emissions are ambiguous and depend on how income changes translate into consumption patterns.

The inter-industry composition effect refers to how trade changes the allocation of production across sectors and countries, shifting the geographical distribution of emissions. As shown in Figure 1, specialization based on comparative advantage can either increase or reduce emissions, depending on whether it favors emissions-intensive or low-emissions activities. Beyond trade in final goods, comparative advantage also drives fragmentation through global value chains, supported by advances in logistics and digitalization. Intermediate goods now account for roughly 50%–60% of global trade.^30^ While these patterns appear at the sector level, they are largely driven by firm-level decisions, such as outsourcing and offshoring production stages. Although cost-efficient for firms, this process redistributes emissions across borders.^31^ Environmental regulation plays a central role. When regulatory stringency differs across countries, firms might relocate pollution-intensive activities toward countries with laxer standards. These dynamics are captured by the pollution haven hypothesis and effect (PHH/PHE) and the pollution offshoring hypothesis (POH).^32^^,^^33^^,^^34^ Trade restrictions tend to dampen inter-industry reallocation, but their net impact on emissions remains ambiguous. Outcomes depend on the interaction between trade and environmental regulation, including the relative emissions intensity of trading partners and whether protectionism substitutes for or complements environmental ambition. Trade policy itself may embed environmental distortions. For instance, Shapiro (2021)^35^ finds that tariffs and non-tariff barriers are systematically lower for CO_2_-intensive industries, effectively subsidizing emissions through trade regimes. However, broader assessments that account for full GHG emissions and domestic fuel taxes suggest that this bias may be substantially lower or even absent.^36^

Third, the intra-industry composition effect captures productivity growth driven by firm selection and efficiency gains. As trade liberalization increases competition, less productive firms may exit the market, while more productive firms expand their market share, as described by the Melitz model.^37^ As Cherniwchan et al. (2017) note,^12^ lower average emissions may, therefore, reflect cleaner, more productive firms gaining market share. The pollution reduction by rationalization (PRR) hypothesis similarly suggests that surviving firms are both more efficient and cleaner,^38^ though empirical evidence remains limited. Trade-induced competition can also improve efficiency within firms and support technology adoption,^39^ with productivity gains often, but not always, linked to cleaner processes.^40^ Trade restrictions may weaken these channels, with uncertain implications for both productivity growth and decarbonization.

Finally, the technique effect captures the role of trade in fostering innovation and technological change (Figure 1). Building on Melitz and Redding (2021),^41^ we distinguish two channels: (1) innovation, driven by competition, market expansion, and specialization and (2) diffusion, through knowledge spillovers. Trade and foreign direct investment (FDI) support both channels by enabling the creation and diffusion of new technologies, thereby fostering long-term growth.^42^ Knowledge spillovers occur through vertical linkages (interactions with foreign buyers and suppliers), labor mobility (staff moving from multinationals to domestic firms), and demonstration effects (local firms adopting foreign, often cleaner, practices and technologies). The effectiveness of these spillovers depends on domestic absorptive capacity, including skills, the nature of FDI, and institutional quality.^43^ While FDI can support green innovation, its effects are often context-specific^43^ and technology uptake varies across regions and financial systems.^44^ As a result, the relationship between innovation and emissions—and the extent to which protectionism affects it—depends on broader enabling conditions, as discussed below.

### Interconnections across channels and time

Rather than acting in isolation, the channels discussed earlier—transportation, scale, inter- and intra-industry composition, and technical change—interact in complex and sometimes conflicting ways.

Transport plays a central role in enabling or constraining other channels. Changes in transport costs affect trade volumes, outsourcing decisions, international competition, and thus, domestic production structures and innovation incentives. It, therefore, influences emissions both directly, through fossil fuel use, and indirectly, by shaping how other trade-emission mechanisms unfold. Scale effects are central. Market expansion increases transport activity and emissions, while also reinforcing composition and productivity effects. Larger markets raise returns to innovation and enhance competitiveness in global markets.^45^^,^^46^^,^^47^ Inter-industry specialization reallocates resources toward sectors of varying innovation intensity^41^ and may displace inefficient (and usually dirtier) firms, reinforcing innovation. These feedbacks can generate both reinforcing and offsetting effects. Productivity gains can stimulate innovation by increasing returns to technology adoption, but also lower prices and increase consumption, amplifying scale effects.^48^ Similarly, trade-induced innovation can reduce emissions, for instance through cleaner transport technologies,^49^^,^^50^ but may also involve trade-offs if it diverts resources from other areas of technological progress,^51^ which may in turn have ambiguous effects on emissions. Diffusion spreads innovation gains across firms and regions, reinforcing both productivity and technological change. Trade shocks can disrupt these dynamics: for instance, tariffs may yield short-term benefits but weaken innovation and long-term welfare, complicating their climate implications.^52^

Temporal dynamics further shape these interactions. Some effects are immediate, such as growth in transport emissions. Others unfold more gradually and often involve significant delays, as is the case for innovation, diffusion, and shifts in consumer preferences (Figure 1). These time asymmetries influence long-term emissions trajectories and interact with path dependence and irreversibility. Innovation processes can lock economies into persistent technological pathways, while short-term trade shocks may trigger lasting shifts in production or trade patterns, as illustrated by the efficiency gains observed after the 1970s oil shocks.^53^ Short-term channels such as transport and scale tend to increase emissions, while long-term ones such as innovation and diffusion have more ambiguous or potentially mitigating effects.

### Enablers: Regulations, institutions, politics

Besides temporal dynamics, three additional dimensions—regulatory frameworks, institutional quality, and stakeholder influence—act as enabling conditions shaping the magnitude and direction of trade’s climate impact (Figure 1).

Regulation is a central enabler, particularly through the design and enforcement of environmental policies. It steers innovation and diffusion toward low-carbon technologies, aligning trade-induced innovation with climate goals, though its effectiveness depends on domestic technological capacity.^54^^,^^55^ Regulatory asymmetries across countries can undermine these outcomes by encouraging relocation of polluting activities and carbon leakage (PHE). Harmonization of standards can mitigate this risk.^56^ Trade-related climate instruments—such as carbon border adjustments,^57^^,^^58^^,^^59^ output-based allocations, and cooperative approaches such as climate clubs^60^ or trade-and-climate treaties^61^—may help align competitiveness with decarbonization, though they can also trigger fairness or coordination concerns.^62^

Institutional quality conditions the effectiveness of regulation and the broader ability to translate trade openness into environmental gains. Weak institutions undermine policy credibility and investment signals, locking countries into carbon-intensive paths. By contrast, strong institutions support clean technology development, as well as comparative advantages in clean industries.^63^

Stakeholder influence represents a third critical dimension. Trade instruments are not only economic tools but also political signals. Protectionist measures targeting clean technologies, such as tariffs on electric vehicles or standards for battery production, often aim to foster domestic industries, retain strategic capacity, and build local supply chains, as observed for photovoltaics^64^ and e-mobility. While such measures may raise short-term costs, they can also strengthen political support for the green transition. However, when driven by lobbying from carbon-intensive incumbents, they may entrench polluting industries, delay greener alternatives, and undermine policy credibility and investment incentives.^65^

## Building analytical capacity and designing scenarios for future trade-climate projections

The previous sections outlined how trade influences emissions through multiple, interacting channels, and how their effects vary over time and across contexts. Building on this framework, this section examines whether existing analytical tools are equipped to capture these dynamics and their implications under evolving trade regimes. While sector-specific or partial-equilibrium approaches can provide more detailed insights into specific mechanisms or strategic industries, the discussion below focuses on economy-wide assessments to examine broader systemic interactions and cross-sectoral feedbacks.

Growing protectionism, supply chain reconfiguration, and technological competition raise new challenges by increasing uncertainty around emissions trajectories, reshaping patterns of industrial specialization, and altering the conditions for international cooperation. These developments require analytical approaches that go beyond standard assumptions derived from liberalized trade environments.

We argue that addressing these challenges requires advances along three complementary dimensions: improved data granularity, enhanced modeling, and an expanded scenario space. Together, these elements are needed not only to represent the key channels and enabling conditions identified earlier, but also to explore how structural transformations, such as regionalization, trade fragmentation, or green industrial competition, may reshape emissions pathways.

The following subsections discuss how to strengthen each component within economy-wide assessments that consistently represent production, trade, and emissions to trace the channels linking trade and climate outcomes, with particular relevance for analyzing protectionist regimes where shifting barriers affect production and emissions patterns.

### Improving the granularity and type of available data

**Figure 2:**
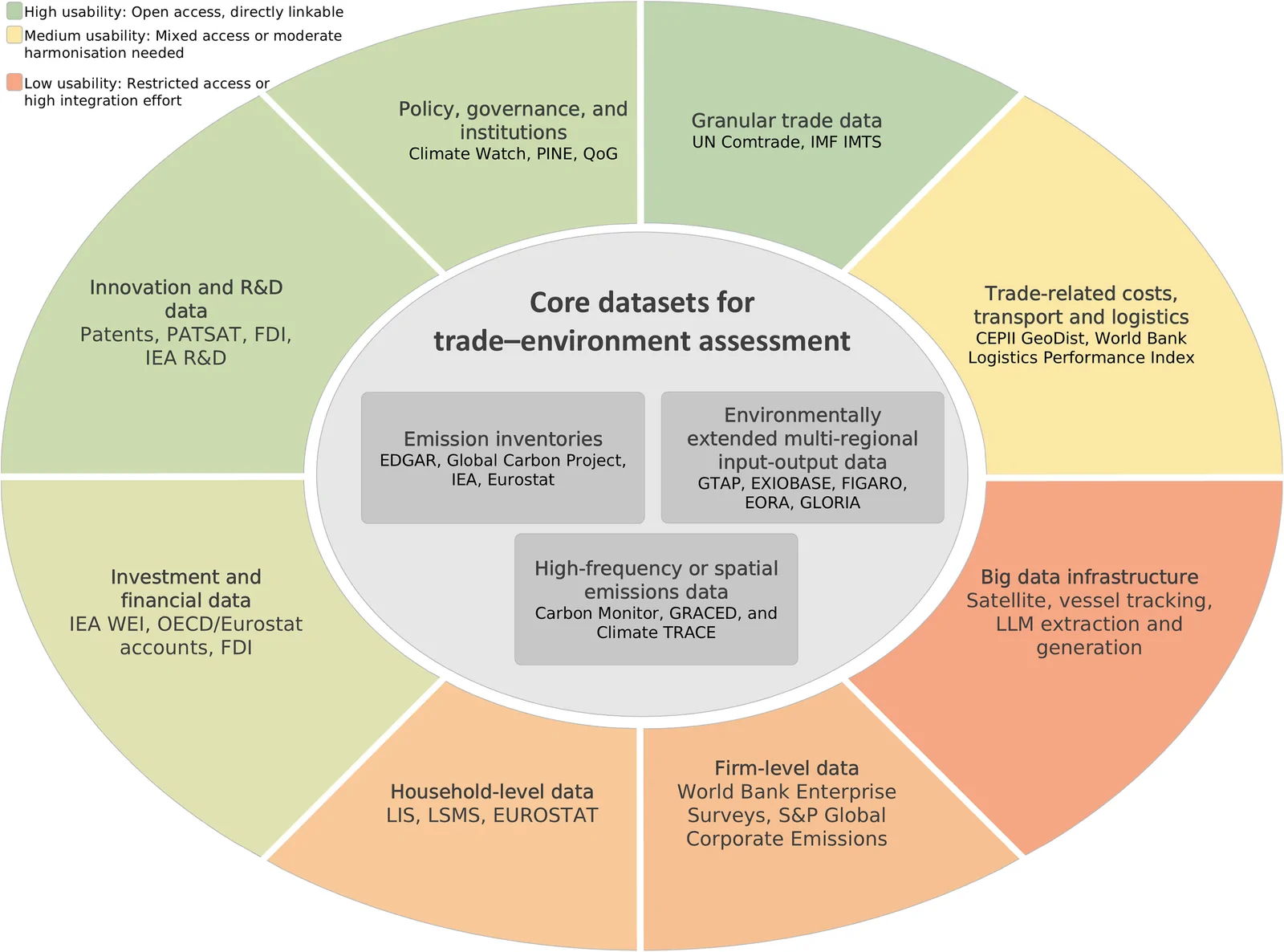
Core datasets currently used for assessing trade-environment linkages (center) and complementary data domains that could enhance macro-micro integration (outer ring) The color intensity of the outer ring reflects the average expert score on data usability, mapped onto a continuous scale from 1 (low) to 3 (high). The rationale and scoring approach are presented in Note S3. Table S2 provides additional discussion of dataset characteristics, methodological differences, limitations, and typical analytical applications.

Numerous public datasets support empirical and modeling work to assess the climate implications of trade. Emission inventories such as Emissions Database for Global Atmospheric Research (EDGAR),^14^ the Global Carbon Project,^13^ and those published by the International Energy Agency (IEA)^15^ and Eurostat^66^ offer historical GHG emissions data across sectors. Newer initiatives such as Carbon Monitor,^16^ Global Gridded Daily CO₂ Emissions (GRACED),^67^ and Tracking Real-Time Atmospheric Carbon Emissions (Climate TRACE)^68^ provide high-frequency or spatial emissions data. Environmentally extended input-output (EEIO) databases—GTAP,^69^ EXIOBASE,^70^ FIGARO-E3,^71^ Eora,^72^ and Global Resource Input-Output Assessment (GLORIA)^73^—offer harmonized multi-regional input-output (MRIO) tables with environmental extensions (Note S1 and Table S2). Despite this richness, important data gaps remain for economy-wide assessments. Figure 2 shows how core datasets can be expanded with complementary data to better capture emerging trade-climate dynamics.

A major gap is the limited integration between granular trade data and macroeconomic datasets, which constrains the tracing of emissions in global value chains.^74^ As shown in Figure 2, detailed trade data—such as UN Comtrade^75^ or the IMF’s International Merchandise Trade Statistics (IMTS)^76^ and International Trade Centre (ITC) Market Access Map (MACMAP)^77^—provide detailed bilateral flows by product (covering over five thousand commodities) and partner (more than 200 economies). Other platforms, such as the World Integrated Trade Solution (WITS),^78^ unify access, yet these data are seldom fully linked to macroeconomic or environmental accounts. This limits the ability to track trade in key technologies and the underlying materials within global value chains, including critical raw materials, batteries, photovoltaic (PV) modules, wind-turbine parts, heat pumps, and electrolysers. Beyond these sources, new big-data infrastructures, such as vessel tracking, bill of lading, and satellite-based sources, further increase spatial and temporal resolution, providing high-frequency, spatially detailed evidence on supply-chain dynamics, transport-related emissions, and trade shocks.^79^^,^^80^^,^^81^ This integration becomes essential in the context of strategic trade policies, where changes in market access and trade diversion reshape production networks and embodied emissions, often at a very granular product level.

A second limitation concerns the representation of trade-related costs, especially transport and logistics, which are often simplified or omitted in EEIO tables and national accounts. These costs shape the carbon intensity of trade and the supply-chain structure. Yet most databases use simplified assumptions, overlooking transport mode (e.g., by using aggregate representation), distance (most models do not explicitly represent bilateral trade costs), and logistics efficiency (often aggregated as part of total costs), limiting analysis of shifting trade patterns.^21^^,^^82^ Complementary sources, such as the Centre d’Études Prospectives et d’Informations Internationales (CEPII) GeoDist dataset^83^ and the World Bank Logistics Performance Index,^84^ could improve the representation of trade costs and transport emissions. International bunkers for shipping and aviation are not reported by trading partner, complicating allocation. Improved transport and logistics coverage is especially relevant for protectionist contexts, where rerouted supply chains and shifting modes of transport may offset emission reductions from lower trade volumes.

Better alignment between firm-level data and sectoral aggregates represented in the national accounts is a promising area for future research.^85^ Firm-level data reveal heterogeneity in emissions intensities and energy use, often masked at aggregate levels.^12^^,^^86^ Linking micro and macro data can improve estimates of abatement costs and policy responses,^87^ and allow capturing heterogeneous firm responses as well as intra-sector responses to trade barriers, exit, restructuring, or localization. Datasets such as the World Bank Enterprise Surveys and S&P Global Corporate Emissions^88^^,^^89^ offer comprehensive firm-level data across countries and sectors, although access remains limited. An important challenge in linking sectoral and firm-level data is the need for harmonization between the two data sources, as the underlying micro and macro datasets often use different classifications.

Another major gap concerns the integration of data on heterogeneous households and related equity indicators. Both trade and environmental policies can have heterogeneous effects across household types, requiring targeted policy solutions.^90^^,^^91^ While data on income and consumption patterns across household types are available in many countries, harmonized multi-regional accounts remain limited. Relevant sources include the Luxembourg Income Study Database (LIS),^92^ World Bank’s Living Standards Measurement Study (LSMS),^93^ and Eurostat’s microdata collections.^94^ Incorporating these microdata allows assessing the distributional implications of protectionist measures, which may differ from liberalization-driven adjustments. For example, tariffs on basic goods (such as energy and food) weigh more on low-income households, while those on items such as solar technologies are more relevant for higher-income groups. While there are numerous country- and region-specific datasets suitable for the distributional analysis of trade policies, a harmonized, publicly available, global household-level dataset that could be readily used for such purposes is still to be developed.

Investment and financial data are often missing or insufficiently detailed in macroeconomic datasets. This limits the ability to assess feasibility, diffusion, and financing of technologies such as electric vehicles, green hydrogen, or heat pumps. In particular, the lack of detailed information on investment by sources and destination sectors constrains insights into composition and sourcing of investments, as most models assumed uniform composition of investment mix/capital goods across sectors. This becomes especially problematic with higher sectoral resolution, when investment goods become much more sector-specific (e.g., solar panels used in solar power generation). Splitting these flows between domestic and foreign adds a further data challenge. Including detail on capital costs, risk, and support mechanisms would improve the analysis of clean-tech manufacturing, trade, diffusion, and trade-climate implications.^15^ Relevant data include the IEA World Energy Investment Datafile,^95^ Organisation for Economic Co-operation and Development (OECD) and Eurostat financial accounts by institutional sector,^96^^,^^97^ and UN Conference on Trade and Development (UNCTAD) or OECD FDI statistics,^98^^,^^99^ which together inform capital allocation and financing patterns across economies. More detailed investment data are essential to trace capital reallocation and the reshoring of clean-tech industries under evolving trade regimes.

Data on innovation are also limited. While the IEA provides R&D expenditure data for energy technologies,^100^ these data mainly cover public investment, with more limited information on private investment.^101^ Tracking knowledge flows is equally important for assessing spillovers, using patent data,^102^^,^^103^ PATSTAT, or FDI data, although the latter often lack sectoral detail.^104^ Improved innovation data would enable a better assessment of how trade restrictions affect cross-border knowledge diffusion and the development of domestic green innovation.

Finally, institutional indicators, including measures of governance quality, provide information on the pace and effectiveness of low-carbon transitions.^105^^,^^106^^,^^107^ Although rarely integrated into trade-environment assessments, complementary datasets such as Climate Watch,^108^ OECD PINE,^109^ and the Quality of Government Environmental Indicators^110^ offer insights into policy, governance, and institutions. Institutional indicators are particularly relevant for distinguishing strategic, climate-aligned protectionism from forms of protectionism that support carbon-intensive incumbents.

### Advancing economy-wide modeling frameworks

A range of modeling approaches has been used to analyze trade-climate interactions, each focusing on different dimensions of the trade-climate nexus. These include engineering and energy system models, which simulate technology-specific mitigation options, particularly in transport and industry^20^^,^^111^; microeconomic and firm-level approaches, such as heterogeneous-firm trade models and econometric analyses of policy responses; and EEIO models, especially MRIO frameworks, widely used to trace embodied emissions, outsourcing effects, and carbon leakage.^70^^,^^74^ Life cycle assessment (LCA) and consumption-based accounting offer complementary product- and demand-based perspectives on emissions.^112^^,^^113^^,^^114^ Economy-wide models, including CGE, macroeconometric models, and IAMs, assess systemic trade-climate effects such as reallocation, innovation, socio-economic impacts, and carbon pricing^3^^,^^22^^,^^115^ (Note S2).

Among these, economy-wide frameworks are best suited to analyze trade-climate links, as they capture direct responses and broader effects from income redistribution, production and trade shifts, inter-sectoral linkages, and competitiveness. While simpler or more stylized models can be useful to isolate specific mechanisms, the discussion here focuses on economy-wide frameworks, given their ability to capture shock propagation and spillovers across sectors and regions. We, therefore, identify a set of key modeling enhancements that could improve the ability of these frameworks to capture trade-climate interactions under evolving trade regimes. These include: (1) the representation of trade and production structures, (2) the treatment of dynamics and uncertainty, and (3) the broader economic and institutional context in which these processes unfold.

A key area of potential improvement is the specification of trade within CGE frameworks. Traditional trade theories assume representative firms and comparative advantage driven by factor endowments; subsequent approaches emphasize firm heterogeneity in productivity and market access.^37^^,^^116^ Building on earlier work on increasing returns, firm entry, and monopolistic competition,^117^ these theories explain how trade liberalization drives intra-industry reallocation and productivity gains. Quantitative and spatial models extend this to show how trade costs and geography shape economies^118^^,^^119^ and how climate change and trade interact.^120^^,^^121^ However, these approaches often prioritize tractability over sectoral granularity, abstracting from detailed industry heterogeneity, input-output linkages, and policy details. Yet, in practice, many CGE models still rely on simplified trade representations, such as the Armington specification, which differentiates goods by origin and treats them as imperfect substitutes, or iceberg costs, which represent non-tariff trade frictions through losses during transit. While widely used, these assumptions constrain the analysis of trade policy shocks and structural adjustment within a rather narrow and specific set of assumptions. Incorporating firm heterogeneity and endogenous market structures allows a richer representation of trade dynamics, including differentiated effects of liberalization or protectionism on firms by size, efficiency, and environmental performance, facilitating a more comprehensive analysis of trade policies.^122^ As shown by Balistreri et al. (2026),^123^ model structure can influence outcomes more than parameter changes, underscoring the need for empirically validated assumptions. Comparative evaluation of alternative trade specifications (e.g., Armington vs. Melitz) within comprehensive model validation and back-casting exercises remains limited. Strengthening supply-side trade modeling is particularly important for analyzing protectionist settings, where market access, competitiveness, and firm selection drive production and emissions outcomes, and where trade-policy spillovers might not be properly captured by standard Armington specifications.

Global value chains have long been studied via input-output and trade-in-value-added approaches,^124^^,^^125^ yet their endogenous dynamics are often only partially represented in macro- or general-equilibrium settings.^126^^,^^127^ Moreover, multinational corporations (MNCs) play a key role in transmitting trade policy shocks and diffusing innovation through FDI, technology transfer, and supply chain integration.^128^ Their behavior involves endogenous mark-ups and cost pass-through that vary with market structure. Facing trade barriers, MNCs may pursue “tariff-jumping” by reallocating production or investment across borders,^129^^,^^130^ shaped by both economic and political factors.^131^ Trade policy measures such as tariffs, subsidies, non-tariff barriers or localization requirements directly interact with these structures, reshaping supply chains and associated emissions. Explicit representation of these mechanisms—linking macroeconomic representation with firm-level and innovation modules—improves the assessment of emissions, competitiveness, and innovation under different trade regimes. Modeling global value chains and MNCs is, therefore, essential to capture tariff-jumping, supply-chain reconfiguration, and cross-border investment shifts that are central to the climate implications of protectionism.

Higher sectoral and technological resolution improves model realism and policy assessment. Several approaches have been used to integrate technological dynamics with economy-wide representation. A top-down structure may allow endogenous switching between technologies based on cost or policy signals.^132^ Bottom-up modules can capture specific sectoral or technological constraints.^133^ Hybrid approaches link CGE and techno-economic models to represent sector-specific constraints.^134^ Linking CGE models with complementary approaches, such as agent-based, techno-economic, or transport models, can improve the representation of behavioral, innovation, and spatial dynamics.^135^ This detail is valuable for analyzing protectionism, where targeted policies affect specific technologies or subsectors.

Temporal dynamics are central to trade-climate interactions, particularly for assessing protectionist measures, where trade-offs unfold over time. Tariffs might initially reduce transport emissions by promoting local production, but near-term capacity constraints or dirtier domestic activities could offset these gains. Over time, outcomes depend on whether domestic production becomes cleaner than displaced imports, and whether global supply chains for inputs are still needed, potentially shifting rather than reducing trade-related emissions. Capturing such dynamics requires models with temporal depth. Existing models range from static to recursively dynamic, with a few intertemporally optimizing structures. While static or myopic models remain popular due to their computational simplicity, enhanced temporal treatments better capture investment responses, endogenous innovation, and learning-by-doing effects.^135^^,^^136^ Many models used to support climate strategies tend to smooth over short-term impacts^137^ and have, therefore, been criticized for their inability to properly examine short-term disruptions and crises.^138^ Expanding the temporal representation of models, especially for innovation and sectoral reallocation, beyond traditional medium- to long-run specification, can enable more accurate evaluation of such effects. This is especially relevant for protectionist settings, where short-term disruptions and delayed adjustment processes contrast with the smoother, long-run efficiency gains typical of liberalization.

Enhancing model robustness and transparency via systematic model intercomparison and ensemble exercises helps identify how structural assumptions, closure rules, and parameter choices affect results.^139^ Comprehensive sensitivity analysis across parameters, shocks, and behavioral elasticities helps map uncertainty and build policy confidence.^140^^,^^141^ Recent developments in artificial intelligence and machine learning enable broader and faster exploration of uncertainty.^142^^,^^143^ As most economy-wide models are primarily *ex ante*, strengthening validation approaches, including back-casting and comparison with historical observations, remains an important methodological challenge. Such exercises are especially relevant for protectionist shocks, whose asymmetric and non-linear impacts across sectors and countries may be masked by structural or parametric assumptions.

Broadening the socio-economic and environmental scope of models is equally important. Representing heterogeneous households and firms is key to assessing the distributional impacts of trade and climate policies, central for equity and political feasibility.^91^ While such features are already incorporated in parts of the CGE and microsimulation literature, their integration remains uneven across economy-wide frameworks. In developing and emerging economies, including informal sectors can markedly change estimated impacts of carbon pricing or trade measures, yet most economy-wide models omit this key component.^144^ Expanding frameworks to account for co-benefits, such as health gains from cleaner air or reduced environmental degradation, would provide more comprehensive welfare assessments.^145^ These elements are particularly relevant for understanding how impacts of trade-climate policies are distributed across agents and for assessing their political acceptability, especially in the context of fragmented trade and climate strategies.

Finally, enabling conditions—regulatory stringency, institutional capacity, and political-economy dynamics—critically shape the performance of trade-climate measures. These factors influence both emissions outcomes and the pace and direction of technological change.^146^ Several modeling strategies can capture these layers. Coupling general-equilibrium models with complementary frameworks can capture sectoral regulation and institutional differences across countries. Enhancing treatment of capital flows, investment risk, and financial constraints can help explain why identical trade or climate policies may lead to different technology adoption patterns across countries. Econometric estimates, such as responsiveness to innovation incentives, can proxy institutional capacity. Above all, scenario-based approaches offer flexibility to explore regulatory and political-economy trajectories,^147^ as discussed in the following section.

**Figure 3:**
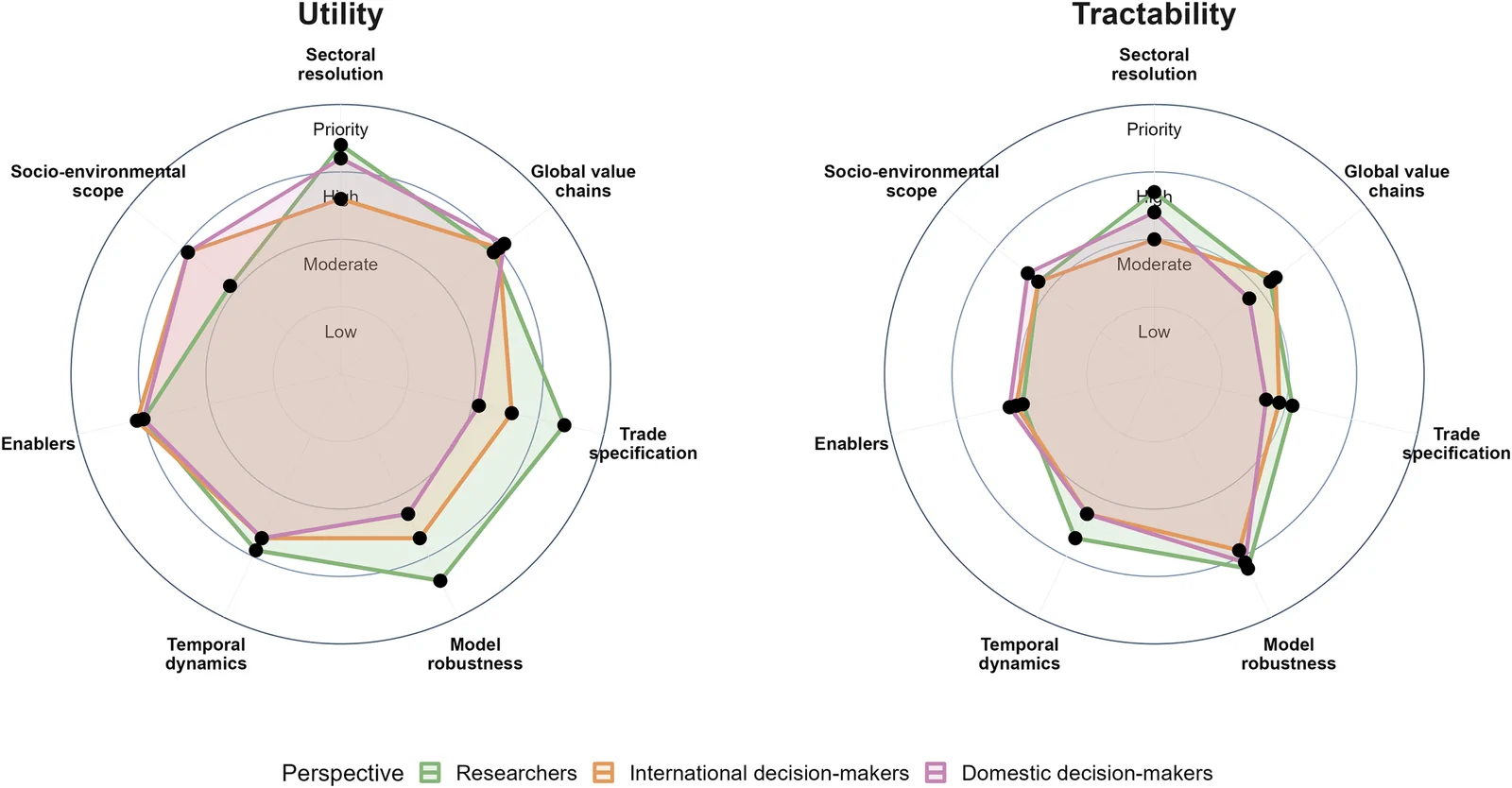
Utility and tractability of model enhancements across perspectives Radar plots compare three perspectives (researchers, international decision-makers, and domestic decision-makers) for the seven proposed model enhancements. Utility and tractability scores are shown on a four-level scale (low, moderate, high, and priority). Filled polygons illustrate how each perspective prioritizes different modeling improvements across the utility and tractability dimensions.

Figure 3 synthesizes assessments of the utility and tractability of the seven key enhancements described earlier from the viewpoints of researchers, international decision-makers, and domestic decision-makers. The rationale and scoring approach are presented in Note S3. Across perspectives, utility is generally assessed to be higher than tractability, highlighting both the importance of these enhancements and the challenges associated with their implementation.

### Expanding the scenario space

Equally important to improving models is how they are used. Scenario design is key for assessing the climate and economic impacts of trade policy. While the scenario literature, including that reviewed by the Intergovernmental Panel on Climate Change (IPCC), spans a wide range of futures, it still lacks explicit treatment of emerging geopolitical and trade dynamics. Other missing dimensions of relevance to trade include human capital/labor,^148^ circular economy,^149^^,^^150^^,^^151^ and different types of extremes.^152^

Targeted trade-focused scenarios can illuminate how trade and climate policy interact. They can enable exploring first-order dimensions that may not be endogenous to models, through “what if” analysis of key drivers. Moreover, better-targeted scenarios can provide common assumptions that can be tested across different modeling tools, leveraging their respective strengths in the climate-trade domain. They can also offer a structured way to explore today’s deep and qualitatively different trade uncertainty, shaped by geopolitical fragmentation, abrupt policy shifts, and potential shocks, through plural and conditional framing rather than single deterministic trajectories.^153^ This expanded scenario space highlights how emerging trade dynamics may increase uncertainty around emissions trajectories and create new challenges for international climate cooperation.

The critical dimensions previously discussed, including environmental ambition, institutional and financial capacity, and political economy, can serve as a foundation for more policy-relevant and trade-focused scenarios. Scenarios often span dimensions and trade-offs using quadrant (2 × 2 matrix) structures.^154^ A well-known example is the Shared Socioeconomic Pathways (SSPs) framework, which organizes future worlds by challenges related to mitigation and adaptation.^155^ While the trade dimension is embedded in the SSPs, and trade barriers are inherently considered in the SSP3 (regional rivalry) and SSP4 (inequality) narratives, the characterization could better represent and quantify the recent paradigm shift on trade and industrial policy. In particular, shifts from globalized trade toward regionalization and increased competition over green technologies may lead to structurally different decarbonization pathways across countries.

**Figure 4:**
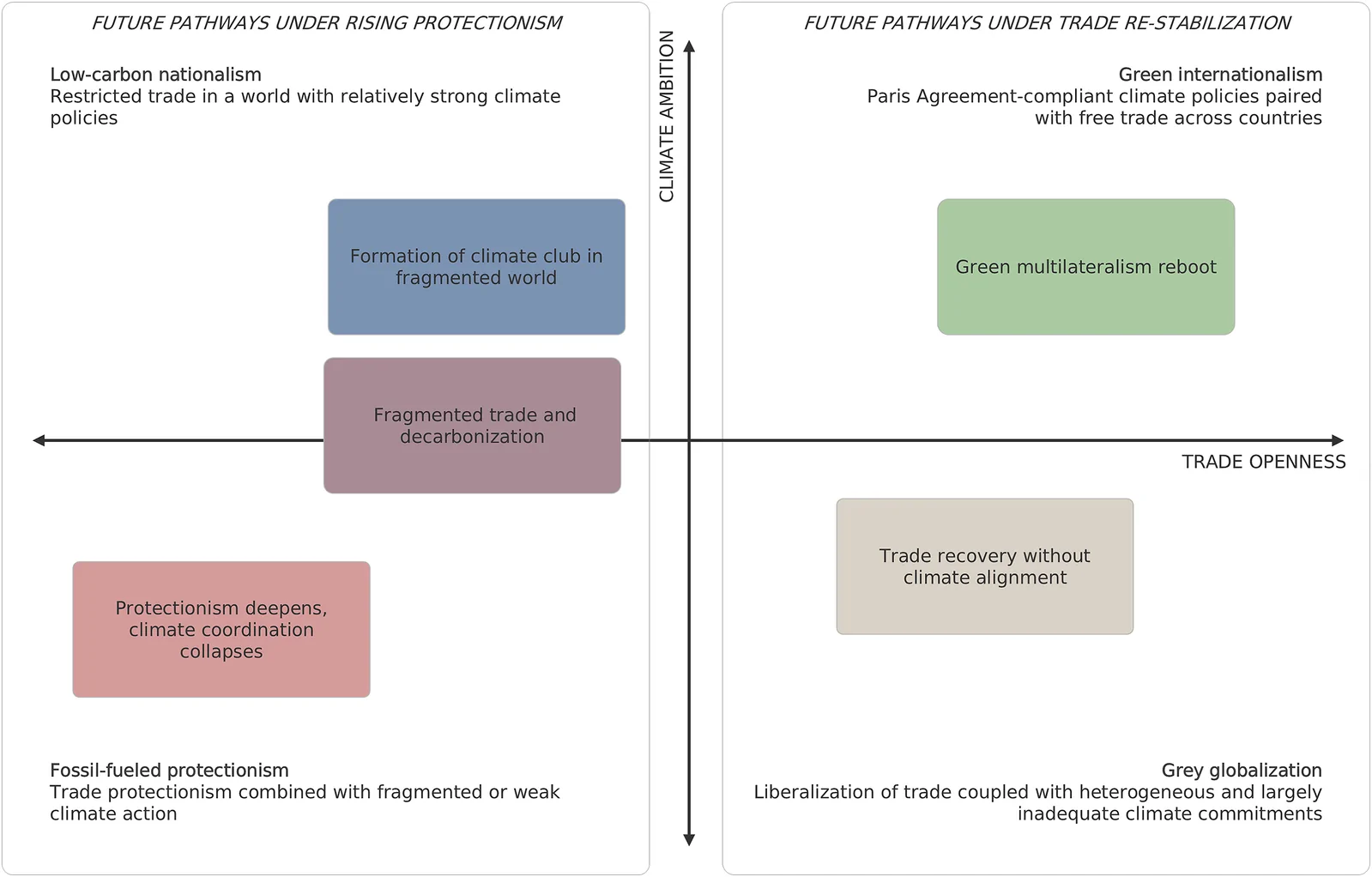
Illustrative scenario framework for examining climate action vis-à-vis trade openness and capturing trade-climate interactions Source: own elaboration.

A similar structure can frame trade-climate scenarios along two axes: trade openness (from protectionism to globalization) and climate ambition (from global inaction to stringent collective climate policy). This spectrum spans four extremes—defined here as green internationalism, low-carbon nationalism, fossil-fueled protectionism, and gray globalization—though arguably more plausible futures lie between these endpoints. Figure 4 illustrates how different combinations of trade openness and climate ambition lead to qualitatively distinct pathways, ranging from cooperative green transitions to fragmented and potentially conflicting strategies.

Additional factors such as institutional capacity and strategic political behavior, already embedded in SSP narratives,^156^ could enrich these scenarios. A structured scenario space would support more robust *ex ante* assessments of how protectionist measures shape long-term emissions and sustainability outcomes. Such scenarios are particularly relevant for anticipating how policy-driven fragmentation may reshape emissions outcomes and the architecture of international cooperation.

## Conclusions and next steps

The relationship between trade and climate outcomes is complex and context-specific, operating through multiple economic channels that interact in non-linear ways. Protectionist measures may raise or lower emissions depending on the dominant channel, context, and the time horizon under consideration. These elements suggest that the trade-climate nexus may increasingly depend on evolving policy regimes and institutional contexts, rather than following the more stable patterns typically associated with liberalized trade environments. They may also increase uncertainty around emissions trajectories and complicate the coordination of climate policies across countries. By mapping the key trade-emissions channels and enablers, and highlighting the data, modeling, and scenario limitations that condition their analysis, this paper provides a foundation for assessing the climate consequences of evolving trade policies and guides improvements in analytical frameworks. Building on these insights, we outline in the following text a set of recommendations for future methodological developments and a forward-looking research agenda.

While existing modeling frameworks have made significant progress in capturing essential aspects of the trade-environment nexus, our analysis suggests that further progress depends on advances along a few key dimensions. First, greater sectoral and commodity detail remains critical*.* Although aggregation bias cannot be entirely avoided in economy-wide models, improving resolution in key areas—such as critical minerals, energy-transition technologies and fuels, emissions-intensive agri-food activities, transport systems, and circular-economy practices—is essential. Achieving this requires stronger efforts to collect and harmonize the underlying data. For example, in critical-minerals supply chains, globally consistent data on extraction and refining profiles across minerals and countries remain limited. The same applies to quantifying the downstream supply chains of energy-transition technologies, such as batteries and electric vehicles. In international transport, detailed data on fossil-fuel use and emissions by commodity and bilateral trade routes are still largely missing, partly due to the bulk nature of these activities. While some high-income countries have made progress in developing such datasets, coverage and quality remain uneven, particularly in developing economies, where future growth is expected to be strongest.

Second, many assessment frameworks still lack sufficient granularity beyond the firm level. This gap spans two complementary dimensions. The first is distributional detail, needed to capture how trade and environmental policies affect different household groups, whose income levels, consumption patterns and exposure to price changes vary widely. The second is spatial detail, reflecting sub-national differences in economic structure, environmental pressures, and policy stringency that can shape the magnitude and distribution of impacts. Combining these dimensions would provide more policy-relevant insights by identifying both *who* is affected and *where* impacts occur. However, harmonized global datasets that integrate detailed household information with spatially resolved accounts remain limited.

Third, further model development is needed to support richer institutional and spatial detail. This includes parametrizing production and consumption processes for new activities and consumer types, identifying substitution possibilities across inputs, consumption categories, and trade options. Doing so requires new research to quantify these relationships through econometric or optimization techniques. Such advances would also support testing alternative model specifications—e.g., different trade mechanisms or demand systems—and their validation via historical or back-casting exercises. Beyond this, the complexity of trade-environment interlinkages often exceeds the scope of a single model, calling for multi-model assessment frameworks. Developing standardized protocols for integrated frameworks would improve comparability and accelerate progress within the broader research community.

Fourth, while data and model developments provide an essential foundation for comprehensive policy analysis, scenario design ultimately shapes analytical rigor and policy relevance. Two areas deserve greater attention. On the one hand, standardized scenarios should better reflect a more fragmented world, where groups of countries follow divergent priorities, some pursuing sustainability, others favoring self-sufficiency. Such divergence may lead to structurally different decarbonization pathways across countries. Although there have been recent advances, widely used frameworks such as the SSPs still tend to assume coherent global development paths. On the other hand, ad hoc analyses should explore more transformative pathways, such as degrowth or absolute resource-decoupling scenarios, to better understand alternative trajectories of the global economy and trade. While such scenarios may have limited short-term policy relevance, they are valuable for mapping the space of possible long-term transitions.

Looking ahead, recent advances in artificial intelligence are likely to reshape analytical frameworks, influencing data collection,^157^ scenario generation,^158^ and the design of modeling frameworks.^159^^,^^160^ Machine learning and adaptive methods could enable models that update as new information becomes available, improving the analysis of complex and evolving trade-climate interactions.

Building on the methodological developments outlined earlier, future research should focus on understanding how institutional quality, regulatory capacity, and political-economy dynamics shape trade-climate interactions: under what conditions do these enabling factors cause protectionist measures to exacerbate environmental risks, and when can they instead support green industrial development? It is also important to analyze how micro- to meso-level behavioral and structural responses—such as consumer preferences, sectoral composition effects, and innovation dynamics—interact with trade policies in a context of rising geopolitical competition and uneven climate ambition, including shifts toward more regionalized trade systems and increasing competition over green technologies, clarifying the conditions under which protectionist measures may yield climate-positive outcomes. At a broader systemic level, further work is needed to understand how trade retaliation, strategic industrial policy, and supply-chain reconfiguration might feedback into emissions, technology diffusion, investment patterns, and international cooperation. These dynamics may contribute to more uncertain and potentially fragmented transition pathways across countries. Advancing this agenda would strengthen the empirical basis for assessing how different trade strategies interact with climate objectives. Ultimately, the question of whether protectionism mirrors liberalization cannot be answered with a simple symmetry argument. While this may hold true for some individual channels, overall outcomes depend on complex interdependencies, temporal asymmetries, and enabling conditions that can generate path-dependent or irreversible effects. These factors suggest that the climate consequences of protectionism are likely to be non-symmetric and context-dependent. Strengthening analytical capacity—through improved data, more robust modeling frameworks, more diverse scenarios, and deeper conceptual and empirical grounding—will be essential to systematically evaluate how different trade strategies interact with climate goals under evolving and uncertain global conditions.

Among the caveats of this perspective, we particularly highlight two. First, its primary scope has been to assess the adequacy of current analytical capabilities to analyze the implications of trade (policy) for climate change and action, rather than the other way around; a different assessment could look at how climate change impacts affect economic systems and trade (policy), distinguishing between different climate impacts (e.g., sea-level rise), their temporal dynamics (e.g., slow- and fast-onset climatic shocks), and their interactions with dimensions that strongly affect trade policy (e.g., migration). Second, trade policy has herein been treated as a contextual driver shaping the mechanisms linking trade and emissions, rather than the primary organizing dimension; future research could instead focus on distinguishing between different climate policy objectives—such as fostering international cooperation (e.g., climate clubs), preventing carbon leakage, or supporting mitigation, adaptation, and/or finance—as this is important for understanding the broader policy landscape.

## Acknowledgments

This work was carried out in the context of the European Commission Horizon Europe project “ENhanced understanding of Trade Impacts on Climate, industries, and the Environment (ENTICE)” (grant no. 101184775). B.B., P.F., L.H., and A.N. also acknowledge support from the European Commission Horizon Europe projects “TRANSIENCE” and “DIAMOND” (grant nos. 101137606 and 101081179, respectively). The sole responsibility for the content of this paper lies with the authors; the paper does not necessarily reflect the opinions of the European Commission or the granting authorities.

## Author contributions

Conceptualization, P.R., V.B., B.B., A.C., M.C., P.F., L.H., and A.N.; funding acquisition, P.R., V.B., B.B., L.C., A.C., M.C., C.F., P.F., L.H., and A.N.; supervision, V.B.; visualization, P.R., V.B., and A.N.; writing – original draft, P.R., V.B., B.B., A.C., M.C., P.F., L.H., and A.N.; writing – review and editing, P.R., V.B., B.B., L.C., A.C., M.C., C.F., P.F., L.H., and A.N. All authors approved the submitted version.

## Declaration of interests

The authors declare no competing interests.

## Declaration of generative AI and AI-assisted technologies in the writing process

During the preparation of this manuscript, the authors used ChatGPT (GPT-4 Turbo, OpenAI) to improve the readability and language of the text. The authors reviewed and edited the content after using the tool and take full responsibility for the final version of the manuscript.

## Supplemental information

Document S1. Tables S1 and S2, Notes S1–S3, and supplemental references
