# Supplementary material for: Trade, protectionism, and climate: Assessing analytical capacity in a changing policy landscape
Source: iScience. 2026 Aug 13;29(9):117132. doi: 10.1016/j.isci.2026.117132 (PMC13495086; doi:10.1016/j.isci.2026.117132)
Supplement: Supplementary file 1 [file mmc1.pdf]

iScience, Volume *29*

Supplemental information

Trade, protectionism, and climate: Assessing analytical capacity in a changing policy landscape

Paola Rocchi, Valentina Bosetti, Baptiste Boitier, Lorenza Campagnolo, Alessia Campolmi, Maksym Chepeliev, Chiara Forlati, Panagiotis Fragkos, Lukas Hermwille, and Alexandros Nikas

SUPPLEMENTAL INFORMATION

Supplemental Table S1. Summary of the links between trade and environment, Related to Figure 1

Channel	Expected emissions effect	Main causal pathway	Main theoretical logic	Typical modelling tools	Typical empirical approaches	Representative references	Indicative observables / metrics	Main links with other channels
International transportation	Usually an increase	Trade expansion raises freight movement across longer distances and transport modes with different carbon intensities	Iceberg trade costs, explicit transport cost functions, spatial trade models	Engineering and energy-system models; transport modules in IAMs; CGE models with transport blocks	Bunker-fuel accounting; mode-shift analysis; scenario analysis; technology case studies	S1–S11	Bunker-fuel CO_2, tonne-km, mode shares, CO_2 per tonne-km, shipping/aviation fuel mix	Affected by scale and preferences; feeds back into trade patterns, outsourcing and offshoring
Scale effect: market size expansion	Usually an increase	Trade enlarges markets and total output	Economies of scale, market-access expansion	Trade models; macro models; partial and general equilibrium models	SDA, IDA, macro-econometric analysis	S12–S14	Output, value added, sectoral scale, emissions per unit output, total emissions	Interacts with composition, productivity and transport
Scale effect: income-induced demand for cleaner goods	Potential decrease, context-dependent	Higher income can shift demand toward cleaner bundles, regulation and abatement	Environmental Kuznets Curve, income-driven preference change	Macro-econometric demand models; reduced-form panel models	Econometric analysis of income-emissions links	S15, S16	Income per capita, consumption shares, emissions intensity of final demand	Linked to composition change and policy preferences
Inter-industry composition: comparative advantage and pollution havens	Ambiguous	Trade reallocates production across sectors and countries with different pollution intensities and regulation	Comparative advantage; pollution haven effect/hypothesis	EE-MRIO; CGE with sector heterogeneity; trade models with endogenous specialization	Sector decomposition; regressions on pollution haven effects	S17, S18	Sector shares, revealed comparative advantage, emissions intensity by sector, regulatory stringency	Driven by scale and preferences; affects productivity and innovation channels
Inter-industry composition: fragmentation and outsourcing in global value chains	Relocation-dependent	Cross-border production slicing redistributes emissions along supply chains	Fragmentation theory, intermediate-goods trade, GVC logic	EE-MRIO; network models; LCA; CBA; macro models with intermediates	Embodied-carbon analysis; territorial vs consumption-based accounting; product LCA with trade data	S19–S25	Embodied CO_2 in trade, foreign value added, import content of exports, supply-chain carbon hotspots	Linked to offshoring, relocation and technology diffusion
Inter-industry composition: MNC offshoring	Relocation-dependent	Multinationals shift stages of production across jurisdictions	Location choice under regulation, costs and	Heterogeneous-firm trade models with location	Firm-level pre/post liberalization analysis; panel	S26	FDI, foreign affiliates, plant relocation,	Closely tied to fragmentation,

Channel	Expected emissions effect	Main causal pathway	Main theoretical logic	Typical modelling tools	Typical empirical approaches	Representative references	Indicative observables / metrics	Main links with other channels
and firm relocation			technology; pollution offshoring	choice; CGE with relocation margins	regressions with emissions and FDI		establishment emissions, sourcing networks	regulation and innovation
Intra-industry composition: firm selection and productivity reallocation	Often a decrease in emissions intensity, net effect context-dependent	Trade reallocates market shares toward more productive firms; least productive firms exit	Melitz-type firm heterogeneity; reallocation effect	Heterogeneous-firm trade models; CGE with firm heterogeneity	Firm-level DiD; exit-entry studies; productivity-emissions matching	S27–S34	Firm TFP, exit rates, exporter status, emissions intensity per firm, within-sector reallocation	Linked to scale, innovation and efficiency gains
Technological progress: innovation	Often a decrease in emissions intensity, but rebound is possible	Trade exposure and competition can stimulate process and product innovation	Endogenous growth; induced innovation; competition effects	Endogenous growth models; trade-innovation models; IAMs with innovation modules	Panel regressions; innovation-emissions studies; policy interaction models	S27, S28, S31–S34	Patents, green patents, R&D, process innovation, energy efficiency, carbon intensity	Reinforces productivity, technology transfer and diffusion
Efficiency gains from trade: technology transfer and innovation diffusion	Usually a decrease in emissions intensity, net effect context-dependent	Firms learn from trade, FDI and global partners; imported knowledge diffuses	Technology diffusion, knowledge spillovers, FDI and learning-by-doing	Dynamic trade models with FDI and spillovers; CGE/IAM hybrids	Firm-level FDI studies; adoption regressions; cross-country diffusion analysis	S35–S37	FDI inflows, imported intermediates, foreign ownership, technology adoption, spillover measures	Connects directly with innovation, productivity and scale

Note: Expected emissions effect indicates the dominant sign under ceteris paribus reasoning; the net effect may remain context-dependent. Representative references are illustrative rather than exhaustive. Broader general-equilibrium channels, including migration, institutions and climate-cooperation mechanisms, are outside the table's explicit scope but may interact with all mechanisms.

Supplemental Table S2. Core environmental and environmentally extended datasets, Related to Figure 2

Database	Type	Scope/Resolution	Typical applications and analytical scope	Source
**EDGAR (v8.0)**	Emission inventory	Global; annual; 1970–2022; 0.1°×0.1° grid; multi-sector, multi-gas; territorial accounting with subnational proxies	Territorial emissions accounting; sectoral and spatial analysis; integration in atmospheric and macro models; not facility-level measurements	S38
**Global Carbon Project (GCP)**	Carbon budget dataset (data family)	Global and national; annual; fossil CO_2 and land-use change; resolution varies by sub-dataset	Global carbon budget estimation; long-term trend analysis; not suitable for high-resolution or near real-time analysis	S5
**IEA CO_2 emissions (energy)**	Energy-emissions statistics	Country-level; annual; sectoral and fuel-level; energy-related CO_2 only (no LULUCF)	Energy-related emissions analysis; policy evaluation; decomposition by sector/fuel; not a full GHG inventory	S39
**Eurostat air emissions accounts (env_ac_ainah_r2)**	Environmental-economic accounts	EU countries; annual; NACE Rev.2 sectors; residence-based accounting	EU cross-country comparisons; linkage with economic accounts; NAMEA-type analysis; not directly comparable to territorial inventories	S40
**Climate TRACE**	Modelled emissions (remote sensing + activity data)	Global; annual (country-level) and monthly (source-level); ~2015–present; asset-level geolocation; sector/subsector detail	Facility-level tracking; identification of large emitters; spatial analysis; uncertainty depends on modelling assumptions	S41
**Global Carbon Atlas**	Data platform (visualization)	Global; mainly country-level annual data; resolution varies across modules	Visualization and communication of global emissions; exploratory analysis; not a primary dataset	S42
**Carbon Monitor**	Near real-time emissions dataset	Near-global; daily; national; 6 sectors; fossil fuel + cement CO_2 only; since 2019	High-frequency analysis; policy and shock tracking; intra-annual dynamics; excludes LULUCF and full GHG coverage	S43
**GRACED**	Gridded emissions dataset	Global; daily; 0.1°×0.1° grid; fossil fuel + cement CO_2; derived from Carbon Monitor + proxies	High-resolution spatial analysis; atmospheric modelling; not suitable for full GHG accounting	S44
**Carbon Monitor Europe**	Near real-time emissions dataset	EU27 + UK; daily; sectoral; fossil CO_2; aviation allocated to departure country	EU monitoring; sectoral dynamics; policy tracking; not consistent with UNFCCC bunker fuel accounting	S45
**Climate Watch**	Data platform (integrated datasets)	Country-level; annual emissions + policy/NDC modules; coverage varies by module	Integrated analysis of emissions, policies, and targets; cross-country comparisons; heterogeneous underlying methodologies	S46
**GTAP (v12)**	Global IO tables + environmental accounts	145 countries + 18 regions; 65 sectors; benchmark years 2004–2023; harmonized global database	CGE modelling; trade–environment analysis; economy-wide policy simulations; resolution depends on release	S47
**EXIOBASE (v3)**	MRIO (EE-MRIO)	44 countries + 5 RoW; 163 industries, 200 products; 1995–2020 (+ nowcasts)	Consumption-based accounting; footprint analysis; GVC analysis; nowcasts introduce additional uncertainty	S22

**OECD ICIO (2023 edition)**	Inter-country IO tables	80 economies; 45 industries; 1995–2022; includes non-OECD countries	Trade in value-added (TiVA); global value chains; environmental extensions; not limited to OECD countries	S48
**FIGARO-E3 (2015 benchmark)**	MRIO (EU-focused)	46 countries + RoW; 176 industries; 213 products; single-year benchmark; multiple extensions (GHG, energy, labor)	High-resolution EU/global trade–environment analysis; footprint studies; consistent with EU statistics	S49
**Eora (latest release)**	MRIO	190 countries; variable sector resolution (full model up to ~15,000 sectors); 1990–2022	Global footprint analysis; broad country coverage; sector detail varies by country; alternative Eora26 provides harmonization	S50
**GLORIA (version-dependent)**	MRIO (SUT-based)	Global; ~160+ regions; ~100–120 sectors; 1990–recent; version-dependent structure	Resource and material footprint analysis; integrated sustainability studies; requires explicit version reference	S51
**PINE (OECD)**	Policy database	150 countries; >4,600 policy instruments; multiple policy types and domains	Analysis of environmental policy instruments and design; not a dataset of emissions or outcomes	S52
**UNSD Environment Statistics**	Indicator system (data family)	Country-level; multiple thematic modules; frequency and coverage indicator-specific	Descriptive environmental statistics; international reporting; requires specification of sub-dataset	S53
**QoG Environmental Indicators (v7)**	Secondary indicator dataset	Country-year panel; mixed temporal coverage; compiled from multiple sources	Empirical analysis linking governance and environment; harmonized dataset; not primary measurement data	S54

Supplemental Note S1. Data sources for emissions analysis, Related to Figure 2 and Table S2

Different data sources are available to analyze trade-climate interactions (Table S2). These resources enable the quantification of emissions across sectors, regions, and time, supporting both historical analysis and scenario design.

Key sources include the Emissions Database for Global Atmospheric Research (EDGAR), which compiles bottom-up emission inventories and integrates them with global activity data to provide detailed sectoral time series[S38], and the Global Carbon Project (GCP), which offers high-resolution assessments of carbon fluxes, including from fossil fuel combustion and land-use change[S5]. Other relevant sources include the International Energy Agency[S39] and Eurostat[S40], which publish energy-related and sectoral emissions data, and platforms like Climate TRACES[S41] and the Global Carbon Atlas[S42], which provide spatial and near-real-time emissions estimates across sectors and regions. These datasets are widely used across the literature discussed in the main text, informing both sector-specific and economy-wide analyses of trade-climate interactions.

Additional sources have emerged to support near-real-time and spatially disaggregated analysis of emissions. Carbon Monitor provides daily estimates of CO_2 emissions from fossil fuel combustion and cement production across major sectors and regions, based on high-frequency activity data[S43]. GRACED (Global Gridded Daily CO_2 Emissions) integrates Carbon Monitor with satellite observations and other sources to offer emissions data at high spatial (0.1° × 0.1°) and daily temporal resolution[S44]. Carbon Monitor Europe extends this approach to deliver daily emissions estimates for EU countries and the UK, disaggregated by sector[S45]. Climate Watch compiles emissions inventories, climate targets, and policy data at the country level, enabling cross-country comparisons and policy tracking[S46].

A range of databases support the integration of environmental indicators into economic and trade analysis. The Global Trade Analysis Project (GTAP) provides harmonized global input-output data with satellite accounts for energy use and emissions, widely used in computable general equilibrium (CGE) modelling[S47]. EXIOBASE offers detailed multi-regional environmentally extended input-output (MRIO) tables, enabling analysis of environmental pressures across sectors and countries[S22]. Similarly, the OECD's Inter-Country Input-Output (ICIO) tables and Eurostat's FIGARO and FIGARO-E3 databases provide harmonized input-output frameworks with environmental extensions[S48,S49,S55]. The Eora database offers high-resolution global input-output tables with environmental extensions, facilitating detailed cross-country analysis[S50]. GLORIA, developed for the UN International Resource Panel, provides time-series MRIO tables with environmental, economic, and social indicators, supporting material footprint and resource use assessments[S51]. These input-output frameworks are complemented by broader indicator databases, including the OECD's Policy Instruments for the Environment (PINE) database[S52], the UN Statistics Division's environment statistics[S53], and the Quality of Government Environmental Indicators Dataset[S54], which support assessments of trade-related environmental impacts.

Supplemental Note S2. Modelling frameworks for emissions analysis, Related to Figure 1

This supplementary note summarizes the main modelling frameworks used to assess the environmental impacts of trade. These include engineering-based and energy system models, environmentally extended input–output approaches, and economy-wide frameworks such as computable general equilibrium, macroeconometric, and integrated assessment models.

Engineering-based and energy system models are typically used to assess technology-specific mitigation options within individual sectors. As such, they are particularly well suited to studying international transportation channels, focusing on mode-specific emissions, energy use, and decarbonization pathways. They can also be applied to examine trade-related shifts in comparative advantage or investment choices within targeted industries—e.g., renewable energy, energy-intensive manufacturing, or clean technology deployment. This category includes engineering optimization systems, bottom-up energy system models, and detailed sectoral modelling tools. Applications have focused on evaluating future emissions trajectories and the performance of technological options across transport modes[S2,S4,S7,S11].

Environmentally extended input–output (EEIO) approaches, including multi-regional input–output (MRIO) models, are widely used to assess emissions embodied in global value chains. By integrating economic transactions with environmental accounts, these models are particularly well suited to analyzing composition effects, including emissions shifts through outsourcing, offshoring, or international specialization. EE-MRIO analysis traces carbon flows across production stages, quantifying embodied emissions in trade and identifying hotspots in supply chains[S22,S25]. Life Cycle Assessment (LCA) offers a complementary, product-based view of emissions, especially when integrated with input–output modelling[S20,S23]. Consumption-based accounting (CBA) assigns emissions to final consumers, highlighting asymmetries between production-based and consumption-based emissions and supporting analysis of industrial activity relocation and carbon leakage[S19,S21]. These approaches are especially useful for capturing the environmental implications of trade reallocation and supply chain restructuring.

Economy-wide models, including Computable General Equilibrium (CGE) models, macroeconometric frameworks, and Integrated Assessment Models (IAMs), expand the analytical scope by embedding trade in broader socio-economic, environmental, and policy systems. These models are well suited to assessing interactions across multiple channels, including scale effects, policy feedbacks, sectoral composition, and, in some cases, innovation dynamics. Partial equilibrium IAMs simulate energy and transport systems with detailed technological pathways, while treating broader economic responses exogenously. They are widely used for global mitigation scenario analysis, including in the IPCC assessments[S6,S8,S10]. In contrast, CGE models capture endogenous trade responses, factor reallocation, and carbon pricing effects across the economy[S1]. While these models provide a systemic lens, many continue to represent trade via simplified assumptions—like iceberg costs and Armington assumptions—and lack explicit treatment of transport emissions, firm heterogeneity, or technology diffusion, limiting their ability to fully capture complex trade–climate interactions[S3].

Supplemental Note S3. Methodological framework for assessing data usability and modelling enhancements, Related to Figures 2 and 3

The assessments reported in Figures 2 and 3 are based on expert judgments pooled across the author team, drawing on complementary expertise in international trade, climate policy, and quantitative economic modelling. They reflect the collective assessment of a small group of experts with direct experience in developing, applying, and interpreting trade–environment models. The resulting scores are not intended to represent statistically robust estimates. As with any expert-based exercise, they involve a degree of subjectivity and depend on the contributors' knowledge and modelling experience. Instead, they provide an indicative synthesis intended to structure discussion of perceived priorities, trade-offs, and implementation challenges.

The two figures rely on related but distinct elicitation approaches, reflecting differences in the nature of the objects being assessed and in the intended level of granularity.

Figure 2 provides an overview of core datasets currently used in trade-environment analysis and complementary data domains that could strengthen macro-micro integration. The assessment underlying this figure follows a qualitative ranking approach. Data domains are classified into three broad usability categories (high, medium, and low) based on expert judgment regarding data accessibility, degree of standardization, and the integration effort required for use in macroeconomic or trade-focused modelling frameworks. High-usability data typically correspond to open-access datasets that are directly linkable to existing model structures. Medium-usability data involve mixed access conditions or require moderate harmonization, while low-usability data are characterized by restricted access, limited coverage, or substantial integration and processing effort.

Figure 3 presents a more structured assessment of modelling enhancements, evaluated along two dimensions, utility and tractability, from three perspectives that frequently shape the development and application of trade-environment models. The researcher perspective represents researchers and modelling practitioners, whose priorities center on methodological coherence, accurate representation of underlying mechanisms, and technical innovation. The international decision-maker perspective reflects the needs of organizations engaged in multilateral or cross-country assessments, where comparability and the exploration of divergent global pathways are central. The domestic decision-maker perspective refers to national policymakers and sectoral stakeholders, who are primarily concerned with feasibility, institutional capacity, and sector-specific implications within domestic economies. These perspectives are intended as illustrative categories that highlight how perceived priorities and constraints may vary across policy and analytical contexts.

The utility dimension captures the perceived relevance of each modelling enhancement for improving the capacity of models to represent key mechanisms discussed in the main text. Higher scores reflect cases where an enhancement is seen as particularly valuable for capturing important features of trade-climate interactions, such as sectoral detail, dynamic adjustment processes, or the treatment of uncertainty. Lower scores indicate cases where the analytical benefit is expected to be more limited or where additional complexity is unlikely to substantially alter policy-relevant insights.

Tractability reflects the perceived feasibility of implementing each enhancement in operational modelling frameworks, taking into account data requirements, methodological readiness, and computational constraints. Enhancements that rely on widely available datasets or established modelling practices tend to score higher, whereas those requiring substantial changes to model architecture, specialized data, or still-emerging techniques tend to receive lower tractability scores.

Across the seven enhancements, the three perspectives show broad convergence in tractability, with assessments clustering within a relatively narrow range between low and moderate. This pattern suggests that feasibility constraints, such as data availability, model structure, and integration requirements, are perceived as broadly similar across analytical, international, and domestic viewpoints.

By contrast, utility scores are, on average, higher than tractability scores, underscoring both the perceived importance and the complexity of advancing these modelling enhancements. Utility also exhibits greater variation across enhancements, reflecting differences in how each improvement is expected to enrich the representation of trade-climate mechanisms. Some enhancements, such as the inclusion of global value-chain structures or institutional and political-economy enablers, are viewed as broadly beneficial across perspectives. Others, such as socio-environmental scope, sectoral disaggregation, or structured scenario and robustness analysis, exhibit more differentiated evaluations, depending on whether the primary emphasis lies on methodological refinement, cross-country comparability, or domestic policy design.

SUPPLEMENTAL REFERENCES

S1. Cristea, A., Hummels, D., Puzzello, L., and Avetisyan, M. (2013). Trade and the greenhouse gas emissions from international freight transport. Journal of Environmental Economics and Management *65*, 153–173. https://doi.org/10.1016/j.jeem.2012.06.002.

S2. Doukas, H., Spiliotis, E., Jafari, M.A., Giarola, S., and Nikas, A. (2021). Low-cost emissions cuts in container shipping: Thinking inside the box. Transportation Research Part D: Transport and Environment *94*, 102815. https://doi.org/10.1016/j.trd.2021.102815.

S3. Felbermayr, G., Peterson, S., and Wanner, J. (2025). Trade and the environment, trade policies and environmental policies—How do they interact? Journal of Economic Surveys *39*, 1148–1184. https://doi.org/10.1111/joes.12628.

S4. Fragkos, P. (2022). Decarbonizing the International Shipping and Aviation Sectors. Energies *15*, 9650. https://doi.org/10.3390/en15249650.

S5. Friedlingstein, P., O'Sullivan, M., Jones, M.W., Andrew, R.M., Hauck, J., Landschützer, P., Le Quéré, C., Li, H., Luijkx, I.T., Olsen, A., et al. (2025). Global Carbon Budget 2024. Earth System Science Data *17*, 965–1039. https://doi.org/10.5194/essd-17-965-2025.

S6. Jaramillo, P., Kahn Ribeiro, S., Newman, P., Dhar, S., Diemuodeke, O.E., Kajino, T., Lee, D.S., Nugroho, S.B., Ou, X., Hammer Strømman, A., and Whitehead, J. (2022). Transport. In Climate Change 2022: Mitigation of Climate Change, P.R. Shukla, J. Skea, R. Slade, A. Al Khourdajie, R. van Diemen, D. McCollum, M. Pathak, S. Some, P. Vyas, R. Fradera, M. Belkacemi, A. Hasija, G. Lisboa, S. Luz, and J. Malley, eds. (Cambridge University Press), pp. 1049–1160. https://doi.org/10.1017/9781009157926.012.

S7. Jonkeren, O., Friso, K., and Hek, L. (2023). Changes in external costs and infrastructure costs due to modal shift in freight transport in North-western Europe. Journal of Shipping and Trade *8*, 24. https://doi.org/10.1186/s41072-023-00154-9.

S8. Müller-Casseres, E., Leblanc, F., van den Berg, M., Fragkos, P., Dessens, O., Naghash, H., Draeger, R., Le Gallic, T., Tagomori, I.S., Tsiropoulos, I., et al. (2024). International shipping in a world below 2 °C. Nature Climate Change *14*, 600–607. https://doi.org/10.1038/s41558-024-01997-1.

S9. Payen, S., Basset-Mens, C., and Perret, S. (2015). LCA of local and imported tomato: an energy and water trade-off. Journal of Cleaner Production *87*, 139–148. https://doi.org/10.1016/j.jclepro.2014.10.007.

S10. Speizer, S., Fuhrman, J., Aldrete Lopez, L., George, M., Kyle, P., Monteith, S., and McJeon, H. (2024). Integrated assessment modeling of a zero-emissions global transportation sector. Nat Commun *15*, 4439. https://doi.org/10.1038/s41467-024-48424-9.

S11. Verschuur, J., Salmon, N., Hall, J., and Bañares-Alcántara, R. (2024). Optimal fuel supply of green ammonia to decarbonise global shipping. Environ. Res.: Infrastruct. Sustain. *4*, 015001. https://doi.org/10.1088/2634-4505/ad097a.

S12. Backus, D.K., Kehoe, P.J., and Kehoe, T.J. (1992). In search of scale effects in trade and growth. Journal of Economic Theory *58*, 377–409. https://doi.org/10.1016/0022-0531(92)90060-U.

S13. Cherniwchan, J., Copeland, B.R., and Taylor, M.S. (2017). Trade and the Environment: New Methods, Measurements, and Results. Annual Review of Economics *9*, 59–85. https://doi.org/10.1146/annurev-economics-063016-103756.

S14. Lindmark, M. (2004). Patterns of historical CO2 intensity transitions among high and low-income countries. Explorations in Economic History *41*, 426–447. https://doi.org/10.1016/j.eeh.2004.01.003.

S15. Dinda, S. (2004). Environmental Kuznets Curve Hypothesis: A Survey. Ecological Economics *49*, 431–455. https://doi.org/10.1016/j.ecolecon.2004.02.011.

S16. Kukla-Gryz, A. (2009). Economic growth, international trade and air pollution: A decomposition analysis. Ecological Economics *68*, 1329–1339. https://doi.org/10.1016/j.ecolecon.2008.09.005.

S17. Levinson, A., and Taylor, M.S. (2008). Unmasking the Pollution Haven Effect. International Economic Review *49*, 223–254. https://doi.org/10.1111/j.1468-2354.2008.00478.x.

S18. Taylor, M.S. (2005). Unbundling the Pollution Haven Hypothesis. Advances in Economic Analysis & Policy *4*. https://doi.org/10.2202/1538-0637.1408.

S19. Davis, S.J., and Caldeira, K. (2010). Consumption-based accounting of CO2 emissions. Proceedings of the National Academy of Sciences *107*, 5687–5692. https://doi.org/10.1073/pnas.0906974107.

S20. Miettinen, P., and Hämäläinen, R.P. (1997). How to benefit from decision analysis in environmental life cycle assessment (LCA). European Journal of Operational Research *102*, 279–294. https://doi.org/10.1016/S0377-2217(97)00109-4.

S21. Peters, G.P., Minx, J.C., Weber, C.L., and Edenhofer, O. (2011). Growth in emission transfers via international trade from 1990 to 2008. Proceedings of the National Academy of Sciences *108*, 8903–8908. https://doi.org/10.1073/pnas.1006388108.

S22. Stadler, K., Wood, R., Bulavskaya, T., Södersten, C.-J., Simas, M., Schmidt, S., Usubiaga, A., Acosta-Fernández, J., Kuenen, J., Bruckner, M., et al. (2018). EXIOBASE 3: Developing a Time Series of Detailed Environmentally Extended Multi-Regional Input-Output Tables. Journal of Industrial Ecology *22*, 502–515. https://doi.org/10.1111/jiec.12715.

S23. Tukker, A., and Dietzenbacher, E. (2013). Global Multiregional Input–Output Frameworks: An Introduction and Outlook. Economic Systems Research *25*, 1–19. https://doi.org/10.1080/09535314.2012.761179.

S24. Vasudevan, S., and Manalaya, S.B. (2023). Trade Continuity and Global Production Sharing in Emerging Economies: Evidence from Panel Gravity Analysis. The International Trade Journal *37*, 571–594. https://doi.org/10.1080/08853908.2022.2072416.

S25. Wiedmann, T., and Lenzen, M. (2018). Environmental and social footprints of international trade. Nature Geosci *11*, 314–321. https://doi.org/10.1038/s41561-018-0113-9.

S26. Choi, J., Hyun, J., Kim, G., and Park, Z. (2023). Trade Policy Uncertainty, Offshoring, and the Environment: Evidence from US Manufacturing Establishments. IZA Discussion Paper No. 15919 (IZA Institute of Labor Economics). https://docs.iza.org/dp15919.pdf.

S27. Demircan Çakar, N., Gedikli, A., Erdoğan, S., and Yıldırım, D.Ç. (2021). A comparative analysis of the relationship between innovation and transport sector carbon emissions in developed and developing Mediterranean countries. Environ Sci Pollut Res Int *28*, 45693–45713. https://doi.org/10.1007/s11356-021-13390-y.

S28. Feng, L., Shi, Y., Yang, Z., Lam, J.F.I., Lin, S., Zhan, J., and Chen, H. (2025). Dynamic correlation of environmental regulation, technological innovation, and corporate carbon emissions: empirical evidence from China listed companies. Sci Rep *15*, 8433. https://doi.org/10.1038/s41598-025-92456-0.

S29. Kreickemeier, U., and Richter, P.M. (2014). Trade and the Environment: The Role of Firm Heterogeneity. Review of International Economics *22*, 209–225. https://doi.org/10.1111/roie.12092.

S30. Melitz, M.J. (2003). The Impact of Trade on Intra-Industry Reallocations and Aggregate Industry Productivity. Econometrica 71, 1695–1725. https://doi.org/10.1111/1468-0262.00467.

S31. Melitz, M.J., and Redding, S.J. (2021). Trade and Innovation. NBER Working Paper No. 28945. https://doi.org/10.3386/w28945.

S32. Su, L. (2025). Environmental regulation and corporate green innovation: evidence from the implementation of the total energy consumption target in China. Journal of Business Economics 95, 499–526. https://doi.org/10.1007/s11573-024-01207-6.

S33. Wu, Y., Zhang, M., and Wang, Z. (2024). Technological innovation and environmental performance: evidence from China. Industrial Management & Data Systems *124*, 1746–1768. https://doi.org/10.1108/IMDS-11-2023-0804.

S34. Yu, D., Soh, W., Amin Noordin, B.A., Dato Haji Yahya, M.H., and Latif, B. (2022). The impact of innovation on CO2 emissions: The threshold effect of financial development. Front. Environ. Sci. *10*. https://doi.org/10.3389/fenvs.2022.980267.

S35. Keller, W. (2004). International Technology Diffusion. Journal of Economic Literature *42*, 752–782. https://doi.org/10.1257/0022051042177685.

S36. Santos, E. (2023). FDI and Firm Productivity: A Comprehensive Review of Macroeconomic and Microeconomic Models. Economies *11*, 164. https://doi.org/10.3390/economies11060164.

S37. Smarzynska Javorcik, B. (2004). Does Foreign Direct Investment Increase the Productivity of Domestic Firms? In Search of Spillovers Through Backward Linkages. American Economic Review *94*, 605–627. https://doi.org/10.1257/0002828041464605.

S38. Ferrario, F.M., Crippa, M., Guizzardi, D., Muntean, M., Schaaf, E., Vullo, E.L., Solazzo, E., Olivier, J., and Vignati, E. (2019). EDGAR v5.0 Greenhouse Gas Emissions (European Commission, Joint Research Centre). https://doi.org/10.2905/JRC.7ANG4XG.

S39. International Energy Agency (2023). CO2 Emissions in 2023: A new record high, but is there light at the end of the tunnel? IEA. https://www.iea.org/reports/co2-emissions-in-2023/executive-summary.

S40. Eurostat (2024). [env_ac_ainah_r2] Air emissions accounts by NACE Rev. 2 activity (Eurostat database). Eurostat Database. https://ec.europa.eu/eurostat/databrowser/view/env_ac_ainah_r2/default/table?lang=en (accessed July 16, 2026).

S41. Climate TRACE Coalition (2023). Emissions data by sector and facility. https://climatetrace.org (accessed July 16, 2026).

S42. Global Carbon Atlas. Carbon Emissions. https://globalcarbonatlas.org/emissions/carbon-emissions/ (accessed July 16, 2026).

S43. Liu, Z., Ciais, P., Deng, Z., Davis, S.J., Zheng, B., Wang, Y., Cui, D., Zhu, B., Dou, X., Ke, P., et al. (2020). Carbon Monitor, a near-real-time daily dataset of global CO2 emission from fossil fuel and cement production. Sci Data *7*, 392. https://doi.org/10.1038/s41597-020-00708-7.

S44. Dou, X., Wang, Y., Ciais, P., Chevallier, F., Davis, S.J., Crippa, M., Janssens-Maenhout, G., Guizzardi, D., Solazzo, E., Yan, F., et al. (2022). Near-real-time global gridded daily CO2 emissions. The Innovation 3, 100182. https://doi.org/10.1016/j.xinn.2021.100182.

S45. Ke, P., Deng, Z., Zhu, B., Zheng, B., Wang, Y., Boucher, O., Arous, S.B., Zhou, C., Andrew, R.M., Dou, X., et al. (2023). Carbon Monitor Europe near-real-time daily CO2 emissions for 27 EU countries and the United Kingdom. Sci Data *10*, 374. https://doi.org/10.1038/s41597-023-02284-y.

S46. World Resources Institute. Climate Watch: Climate Data for Action. https://www.climatewatchdata.org/ (accessed July 16, 2026).

S47. Aguiar, A., Chepeliev, M., Corong, E.L., McDougall, R., and van der Mensbrugghe, D. (2019). The GTAP Data Base: Version 10. Journal of Global Economic Analysis 4, 1–27. https://doi.org/10.21642/JGEA.040101AF.

S48. Organisation for Economic Co-operation and Development (2023). Inter-Country Input-Output tables. OECD. https://www.oecd.org/en/data/datasets/inter-country-input-output-tables.html (accessed July 16, 2026).

S49. Cazcarro, I., Usubiaga-Liaño, A., Román, M.V., Piñero, P., Dietzenbacher, E., Rueda-Cantuche, J.M., and Arto, I. (2025). FIGARO-E3: a high-resolution extended multi-regional input-output database consistent with official statistics. Sci Data *12*, 575. https://doi.org/10.1038/s41597-025-04431-z.

S50. Lenzen, M., Moran, D., Kanemoto, K., and Geschke, A. (2013). Building Eora: A Global Multi-Region Input–Output Database at High Country and Sector Resolution. Economic Systems Research 25, 20–49. https://doi.org/10.1080/09535314.2013.769938.

S51. Industrial Ecology Lab (IELAB) (2021). GLORIA: Global Multi-Regional Input-Output Database. IELAB. https://ielab.info/labs/ielab-gloria (accessed July 16, 2026).

S52. Organisation for Economic Co-operation and Development. Policy Instruments for the Environment (PINE) Database. https://www.oecd.org/en/data/datasets/policy-instruments-for-the-environment-pine-database.html (accessed July 16, 2026).

S53. United Nations Statistics Division. UNSD Environmental Indicators. https://unstats.un.org/unsd/envstats/qindicators (accessed July 16, 2026).

S54. Povitkina, M., Alvarado Pachon, N., and Dalli, C.M. (2021). The Quality of Government Environmental Indicators Dataset, Version Sep21 (The Quality of Government Institute, University of Gothenburg). https://www.gu.se/en/quality-government.

S55. Eurostat (2024). FIGARO tables (2024 edition): annual EU inter-country supply, use and input-output tables. https://ec.europa.eu/eurostat/web/esa-supply-use-input-tables/database (accessed July 16, 2026).
